# Quality of life in patients with pituitary tumors: features of diagnostics

**DOI:** 10.1192/j.eurpsy.2025.1701

**Published:** 2025-08-26

**Authors:** Y. Sidneva, L. Astafyeva, P. Kalinin, A. Shkarubo, M. Kutin, I. Voronina, D. Fomichev, D. Andreev, O. Sharipov, I. Chernov, A. Donskoy, I. Klochkova, I. Badmaeva

**Affiliations:** 1 neuropsychiatry, N.N.Burdenko National Medical Research Institute of Neurosurgery; 2 neurorehabilitation, Clinical and Research Institute of Emergency Pediatric Surgery and Trauma; 3 neuroendocrinology; 4 neurosurgery; 5 neurology, N.N.Burdenko National Medical Research Institute of Neurosurgery, Moscow, Russian Federation

## Abstract

**Introduction:**

The term “quality of life” is widely used in the world community, has an interdisciplinary concept that characterizes the effectiveness of all aspects of human life. But it should be understood that its main component is the psychological and psychiatric part, which is based on self-perception. And in this regard, it is obvious that the degree of quality of life of patients with pituitary tumors, manifested by a complex clinical picture, sometimes a multi-component treatment strategy, is important for understanding, diagnostics and correction.

**Objectives:**

to assess and compare the degree of quality of life in patients with pituitary tumors.

**Methods:**

120 patients (18-79 years old) after surgery for diencephalic tumor, 2019-2022. The following scales were proposed: 1) assessment of the severity of cognitive impairment and social maladjustment - the Global Deterioration Rating (GDR) scale (stages 1 to 7, where 1 is no impairment/deficit); 2) assessment of the general condition of cancer patients - the Karnofsky index (from 0% to 100%, where 100% is normal condition, no complaints); 3) a short version of the quality of life assessment (SF-12, 2000). All patients were examined dynamically by a psychiatrist.

**Results:**

The quality of life was assessed during treatment, when the degree: improves, remains unchanged, worsens. Different dynamics were noted with different tumor morphology, volumes of surgical intervention, and course of recovery after surgery. Thus, after surgery, the number of patients with severe disorders in large and giant tumors, especially with spread to the third ventricle (according to the Karnofsky and GDR scales), increased, respectively, they were found to have a low degree of “quality of life”. The use of questionnaires (SF-12) showed the inappropriateness of their use, since 33-78% of patients (with different tumor morphology) showed personality changes, they were not fully aware of their condition. Assessment by scales revealed the following features: 1) A large sample, no universal; 2) Mostly self-questionnaires, which is questionable due to the high percentage of patients with lack of criticality, inadequate assessment of the situation and themselves; 3) Conducted by different specialists; 4) To interpret the results, an assessment by a psychiatrist is necessary. Patients with pituitary tumors were divided into: 1) with pronounced health problems - sufficient quality of life; 2) with minimal symptoms - deeply “unhealthy”.

**Conclusions:**

In assessing the “quality of life”, the primary factors are not the symptoms of the disease and their manifestations, but the perception of oneself and the ability to feel “happiness”. In the case of pituitary tumors, non-specific scales and self-questionnaires should be used with reservations; for the adequacy of the interpretation of the results, it is worth comparing the data with the psychiatric report.

**Disclosure of Interest:**

None Declared

